# Safe Meat, Smart Science: Biotechnology’s Role in Antibiotic Residue Removal

**DOI:** 10.3390/antibiotics14121264

**Published:** 2025-12-15

**Authors:** Jovana Novakovic, Isidora Milosavljevic, Maria Stepanova, Galina Ramenskaya, Nevena Jeremic

**Affiliations:** 1 Department of Pharmacy, Faculty of Medical Sciences, University of Kragujevac, 69 Svetozara Markovica St., 34000 Kragujevac, Serbia; jovana.novakovic@fmn.kg.ac.rs (J.N.); isidora.milosavljevic@fmn.kg.ac.rs (I.M.); 2 Center of Excellence for Redox Balance Research in Cardiovascular and Metabolic Disorders, Faculty of Medical Sciences, University of Kragujevac, 34000 Kragujevac, Serbia; 3 A.P. Nelyubin Institute of Pharmacy, Sechenov First Moscow State Medical University, 119991 Moscow, Russia; stepanova_m_yu@staff.sechenov.ru (M.S.); ramenskaya_g_v@staff.sechenov.ru (G.R.)

**Keywords:** food safety, high throughput screening, biosensors, antibiotic residues, phage therapy

## Abstract

The widespread use of antibiotics in livestock farming has led to the persistent issue of antibiotic residues in meat products, raising significant concerns for food safety and public health. These residues can contribute to the emergence and spread of antimicrobial resistance (AMR), a growing global health threat recognized by the World Health Organization. While some regulatory bodies have imposed restrictions on non-therapeutic antibiotic use in animal agriculture, inconsistent global policies continue to hinder unified efforts to reduce AMR risks. This review explores the role of biotechnology in addressing this challenge by offering innovative tools for the detection, degradation, and removal of antibiotic residues from meat. Biotechnological approaches include the use of biosensors, high-throughput screening, enzymatic degradation, microbial bioremediation, genetically engineered bacteria, phage therapy, and phytoremediation. In addition, enabling technologies such as genomics, metagenomics, bioinformatics, and computational modeling support the rational design of targeted interventions. We further examine the integration of these biotechnological strategies within the broader “One Health” framework, which emphasizes the interconnectedness of human, animal, and environmental health. Case studies and recent applications demonstrate the potential of these methods to ensure safer meat production, reduce public health risks, and enhance consumer trust. By focusing on scalable, science-driven solutions, biotechnology offers a promising path toward mitigating antibiotic residues in the food supply and combating the long-term threat of AMR.

## 1. Introduction

The presence of antibiotic residues in meat products is a growing global problem, resulting from the widespread use of antibiotics in livestock farming [1]. Antibiotics are often given to animals not only for therapeutic purposes, but also to promote growth and prevent disease, often leading to the accumulation of antibiotic residues in edible tissues [2,3]. In the United States, it is estimated that up to 80% of all antibiotics sold are used in livestock farming, raising serious concerns about both the presence of antibiotic residues in the food chain and the potential development of antimicrobial resistance (AMR) in the human population [4,5]. More importantly, this practice contributes to the global increase in AMR, which has been declared one of the ten greatest global public health threats by the World Health Organization (WHO) [6].

Antibiotic residues in edible tissues can select for resistant bacterial populations, allowing antimicrobial-resistant strains, such as *Salmonella*, *Escherichia coli*, *Clostridium perfringens*, and *Listeria monocytogenes*, to persist throughout the meat production chain [7,8,9,10,11]. These resistant pathogens pose a unique foodborne threat, as infections caused by resistant strains often require longer treatment and second-line antibiotics, and are associated with increased morbidity [9,10].

Regulatory approaches differ substantially across regions. The European Union has implemented strict regulations prohibiting the non-therapeutic use of antibiotics in livestock, whereas the United States allows certain uses under the US Food and Drug Administration, creating inconsistencies in global mitigation efforts [11]. Given the interconnectedness of human, animal, and environmental health, particularly in the context of antimicrobial resistance and addressing antibiotic residues in the food chain, the need to adopt a comprehensive “One Health” strategy that relies on collaborative, cross-sectoral strategies based on scientific innovation is essential [12].

Foodborne exposure to antimicrobial-resistant bacteria represents not only a direct public health risk but also a significant global economic burden, affecting over 600 million people worldwide, resulting in 420,000 deaths each year [13,14]. For example, in Sweden, the combined direct and indirect costs associated with five major pathogens—including *Campylobacter*, *Salmonella*, *Yersinia*, and *Shigella*—exceed EUR 142 million annually, with *Campylobacter* alone accounting for around EUR 98 million [15]. In France, a study covering the years 2008–2013 estimated that these foodborne pathogens cause between 1.28 and 2.23 million cases of illness annually, resulting in 16,500–20,800 hospitalizations and approximately 250 deaths. The three leading contributors to both illnesses and hospitalizations include *Campylobacter*, nontyphoidal *Salmonella*, and norovirus, while *Salmonella* spp. and *Listeria monocytogenes* are the primary causes of foodborne-related fatalities [16]. Vulnerable groups, including children under five years of age, pregnant women, the elderly, and immunocompromised individuals, are disproportionately affected, with children alone accounting for nearly 40% of all foodborne-related deaths [17,18].

Resistant infections contribute to increased healthcare cost, prolonged hospital stays, productivity losses, and trade limitations, highlighting the urgent need for innovative interventions at the agricultural source. Biotechnology-based tools, including rapid screening, enzymatic degradation, microbial bioremediation, and biosensor-based detection methods, offer precise and scalable approaches to mitigating AMR in the food supply [13]. When combined with responsible antibiotic-use practices in livestock, they form a comprehensive strategy to protect both public health and food security.

The aim of this review is to synthesize current biotechnological approaches for detecting and eliminating antibiotic residues in meat, evaluating their effectiveness and scalability.

### 1.1. Economic and Social Impact of Antimicrobial Resistance Linked to Antibiotic Use in Livestock

Building on the concerns outlined in the Introduction regarding the rise of AMR due to antibiotic use in livestock, it is clear that AMR represents a significant economic and societal burden worldwide. Resistant infections often result in treatment failures, prolonged hospital stays, and the need for more expensive or combination therapies, substantially increasing direct healthcare costs [19,20,21,22]

A significant proportion of AMR in humans is linked to antibiotic residues and resistant bacteria transmitted through meat from intensive farming systems. Infections with resistant strains are associated with higher medical expenditures and longer recovery periods compared to susceptible infections [21,22]

Beyond direct healthcare costs, AMR causes indirect economic losses, including reduced labor productivity due to illness, increased mortality, and trade restrictions on animal products due to safety concerns [23,24]. Low- and middle-income countries are disproportionately affected, as limited resources and surveillance capacity exacerbate both the spread of resistance and its economic impact [21,25].

Investing in prudent antibiotic use in livestock, combined with biotechnological strategies for early detection and elimination of residues in meat, can mitigate these economic consequences. Reducing the incidence of resistant infections at the source not only improves public health outcomes but also represents an economically prudent approach for sustainable food systems [20,21].

### 1.2. Key Challenges in Food Safety Related to Antibiotic Residues and AMR

Addressing the challenges of food safety requires addressing several critical issues, including the globalization of food supply chains, the emergence of new antibiotic-resistant pathogens, and the impact of climate change. The increasing complexity of global food supply chains means that food products often travel long distances, crossing multiple countries before reaching consumers. This complexity increases the risk of contamination at different stages of production, processing, and distribution. The presence of harmful bacteria on food surfaces further increases the risk of cross-contamination, leading to foodborne illness [26].

The emergence of new antibiotic-resistant pathogens poses a significant threat to food safety worldwide. AMR occurs when bacteria, viruses, fungi, and parasites change over time and become less susceptible to drugs, making infections more difficult to treat and increasing the risk of disease spread, serious illness, and death.

Climate change affects food safety by altering the geographic distribution of pathogens and increasing the risk of contamination by natural toxins. Rising temperatures and changing rainfall patterns can create favorable conditions for the growth and spread of bacteria, viruses, and fungi in crops and livestock. Although food is intended to nourish and maintain health, when contaminated or improperly handled, it can cause harm, leading to foodborne illness [27].

## 2. Antibiotic Residues in Meat: Source, Health Risk, and Food Safety Implications

In many developing countries, the use of antibiotics in livestock farming often occurs without proper veterinary supervision, and a large proportion of antibiotics used in animals are not fully metabolized, leading to the accumulation of residues in tissues such as the muscle, liver, and kidney [9]. This can lead to the overuse and misuse of these drugs, and consequently to the presence of residues in food products. In addition, contaminated food and water are also important sources of exposure, contributing to the accumulation of antibiotic compounds in animal tissues. The routine inclusion of sub-therapeutic doses of antibiotics in food or water to promote growth and body weight gain has been identified as a major driver of antibiotic resistance [28]. Inadequate animal husbandry practices and failure to observe appropriate withdrawal periods before slaughter further exacerbate the problem.

The presence of antibiotic residues in meat poses several health risks to consumers. One of the most serious concerns is the promotion of antibiotic resistance in bacteria, making it difficult to treat common infections [29]. Chronic exposure to low levels of antibiotics through food can alter the gut microbiota, impair immune responses, and promote the emergence of resistant bacterial strains, which can then spread systemically or be transmitted to others. Such infections can require more aggressive and expensive treatments, result in prolonged hospital stays, and increase mortality rates. Furthermore, some individuals may experience allergic reactions to certain antibiotics, ranging from mild skin irritation to severe and potentially life-threatening anaphylaxis [1,8,11].

As global demand for animal protein increases, the livestock industry is faced with the challenge of improving productivity while addressing the critical issue of antimicrobial resistance, particularly in intensive production systems such as broiler farming. Such residues can cause allergic reactions and disrupt the physiological balance of the gut microbiota in humans [30]. This not only threatens human health but also affects animal health by increasing morbidity and mortality rates in livestock [31]. Balancing efficiency with sustainability has never been more necessary and challenging.

### 2.1. Prudent Antibiotic Use and Antimicrobial Stewardship in Livestock

The presence of antibiotic residues in meat poses a direct risk to consumer health and contributes to the development of antimicrobial resistance. A substantial proportion of AMR in livestock production arises from non-therapeutic, prophylactic, and otherwise improper antibiotic use. While biotechnological interventions and advanced residue-detection systems are powerful, improving antibiotic-use practices remains the most accessible and cost-effective strategy for reducing AMR, particularly in low- and middle-income countries [21]. Prudent-use programs, including restricting non-therapeutic use, enforcing veterinary oversight, implementing treatment records, improving farm hygiene and biosafety, increasing vaccination coverage, and enhancing farmer education, have consistently demonstrated the ability to reduce antibiotic consumption without compromising animal health or productivity [32,33] (OIE, 2021; EMA, 2023). Existing stewardship frameworks, such as the “5R” principles (Responsibility, Reduction, Replacement, Refinement, and Review) and the “3R” strategies (Reduce, Replace, and Refine), provide practical guidance for judicious antimicrobial deployment and can be adapted to diverse production systems worldwide [34,35].

Non-biotechnological interventions, including vaccination programs, biosecurity measures, optimized nutrition, and improved housing and hygiene, can significantly reduce disease incidence, thereby minimizing the need for antibiotic administration [36,37]. Implementing these measures requires minimal infrastructural investment and can be scaled effectively even in countries with limited resources.

Strengthening responsible antibiotic use therefore represents a practical and scalable component of AMR mitigation, complementing biotechnology-based approaches and serving as an essential foundation for sustainable livestock production systems worldwide.

### 2.2. Challenges in Complying with Regulatory Standards and Guidelines

International and national regulatory bodies have set maximum residue limits for various antibiotics in different types of meat. These standards aim to protect public health by ensuring that antibiotic residues in food remain below harmful levels [38]. Many countries also have regular monitoring programs to verify compliance with these regulations. However, the effectiveness of these regulations varies considerably between regions. In countries with weak regulatory oversight, enforcement of the standards is often inconsistent or absent [39].

The European Union enforces some of the world’s strictest policies, including a complete ban on the non-therapeutic use of antibiotics since 2006 [40,41]. More recently, the EU adopted Regulation (EU) 2022/1255, which further restricts the use of medically important antimicrobials in veterinary medicine to preserve their clinical efficacy [42]. Additionally, the EU maintains harmonized maximum residue limits (MRLs) and comprehensive surveillance mechanisms such as the Rapid Alert System for Food and Feed (RASFF) and the European Surveillance of Veterinary Antimicrobial Consumption (ESVAC) [33].

The United States, on the other hand, follows a different regulatory model under the Food and Drug Administration (FDA), which still permits certain preventive uses of medically important antibiotics under veterinary oversight, as outlined in Guidance for Industry (GFI) documents #213 and #263 [43,44]. Although the U.S. no longer allows growth-promoting use of medically important antibiotics, several drugs remain authorized for disease prevention, and residue limits differ from those set by the EU [45,46]. Consequently, U.S. surveillance programs—such as the National Residue Program (NRP) and the National Antimicrobial Resistance Monitoring System (NARMS)—operate under a separate regulatory and enforcement framework [47,48].

Regulatory diversity extends beyond Europe and North America. Some non-EU countries, including Norway and Switzerland, apply EU-aligned restrictions, while many regions in Latin America and Asia allow broader use of antibiotics in livestock due to limited enforcement capacity or reliance on antibiotics for production efficiency [21,32]. These global discrepancies in policy implementation and enforcement create uneven risk profiles for antibiotic residues, complicate international trade, and challenge harmonized food safety strategies.

Not all antibiotics used in animals are subject to these regulations, leaving potential loopholes that producers can exploit [49]. The intensive and often-uncontrolled use of antibiotics in the agricultural and veterinary sectors highlights the need for improved legislation, effective enforcement, and investment in sustainable alternatives for the prevention of zoonotic and infectious diseases [50].

Given the serious health risks and regulatory challenges posed by antibiotic residues in meat, there is a clear need for innovative strategies to detect and eliminate them. While traditional methods have their place in detecting and eliminating antibiotic residues, they often exhibit limitations in terms of sensitivity, efficiency, and long-term sustainability. In this light, biotechnological solutions stand out as a promising alternative that can significantly improve food safety and reduce dependence on the use of antibiotics in livestock production [51].

## 3. Biotechnology-Enhanced Stewardship and Optimized Antibiotic Use in Livestock

Responsible antibiotic use remains the cornerstone of AMR mitigation; recent biotechnological advancements offer powerful tools to support and enhance prudent antibiotic practices in livestock, particularly in settings where farmers may have limited technical knowledge or resources. These technologies enable real-time monitoring, targeted therapy, and evidence-based decision-making, reducing unnecessary antimicrobial exposure while maintaining animal health and productivity [52].

Biosensors and on-farm diagnostic devices have emerged as practical and cost-effective solutions for early detection of infections or physiological changes in livestock. Unlike traditional laboratory testing, portable electrochemical and optical biosensors can detect specific pathogens, toxins, or biomarkers directly in milk, serum, or fecal samples, allowing veterinarians to adjust treatment protocols only when needed [53]. This approach significantly reduces prophylactic use of antibiotics, which is major driver of AMR in production systems.

Genomic and bioinformatic tools provide additional support for targeted antimicrobial therapy. Whole-genome sequencing of livestock pathogens and metagenomic analysis of farm environments can identify resistance genes and predict antimicrobial susceptibility patterns, enabling veterinarians to select the most valuable antibiotic [54,55]. Similarly, integrated farm management platforms, often leveraging IoT sensors and cloud-based analytics, allow continuous monitoring of animal health indicators (temperature, activity, feed intake) to optimize treatment decisions and reduce unnecessary antibiotic administration [52].

Biotechnology provides alternatives that directly reduce the need for antibiotics, including probiotic formulations, bacteriophage therapies, competitive-exclusion cultures, and microbiome-modulating feed supplements that help stabilize gut flora and enhance immunity [56]. These tools play a crucial role in improving herd health, decreasing disease incidence, and thereby lowering antimicrobial consumption.

These biotechnology-enhanced strategies complement traditional stewardship measures such as vaccination, improved biosecurity, and farmer education. By combining low-cost, accessible management practices with advanced technological support, livestock producers can implement a scalable, practical One Health approach that minimizes AMR risk while ensuring food safety and productivity, even in low- and middle-income countries.

### 3.1. Biotechnological Innovations in Detecting and Eliminating Antibiotic Residues in Meat

Biotechnology represents a promising innovative alternative to conventional methods, offering more sensitive, efficient, and sustainable solutions for the detection and elimination of antibiotic residues in meat [57].

Several biotechnological methods are currently being investigated to address these challenges. These include biosensors for real-time detection and high-throughput screening (HTS) methods for the identification of effective biological agents.

Biotechnological approaches, including enzymatic degradation, microbial bioremediation, and phage therapy, offer targeted and efficient methods for the removal of antibiotic residues from meat products. These methods offer advantages such as increased efficiency, reduced environmental impact, and the ability to act on a broad spectrum of antibiotic compounds. Integrating innovative approaches to meat processing and production can promote safer and more sustainable models that meet the growing consumer demand for high-quality, antibiotic-free meat [58].

The use of genetic editing tools such as CRISPR-Cas systems in livestock offers the potential to improve disease resistance and reduce the overall need for antibiotics [59]. Such advances have the potential to transform the meat industry by improving animal health, meat product quality, and consumer confidence. While the need to reduce antibiotic use in livestock farming is clear, this must be carried out with great care, as it is not possible to completely ban the use of antimicrobials in this area [60].

#### Biotechnological Methods for the Detection of Antibiotic Residues: Biosensors and High-Throughput Screening (HTS)

Biotechnological methods are rapidly transforming techniques for antibiotic detection in meat products. Biosensors and HTS enable early detection and monitoring of residues throughout the food chain.

Biosensors have emerged as promising tools for the rapid and sensitive detection of antibiotic residues in complex food matrices such as meat. These devices are suitable for use in all stages of the food production process, from the farm to the processing plant, because they are very sensitive and specific and can provide results in real time. In comparison to traditional methods such as chromatography or ELISA, biosensors offer faster response times, minimal sample preparation, and potential for miniaturization and on-site deployment, making them ideal for real-time monitoring in the meat production chain [61,62].

Biosensors combine a biological recognition element (enzyme, antibodies, or nucleic acids) with a transducer that converts the biological response into a measurable signal. For example, enzyme-based biosensors quantify antibiotic residues by measuring enzyme inhibition. On the other hand, aptamer-based biosensors use aptamers, single-stranded DNA or RNA molecules that bind to specific target molecules, to capture and detect antibiotic residues. Similarly, immunosensors, another type of biosensor, rely on antibody–antigen interactions to detect residues [63]. An overview of different types of biosensors and their key characteristics is presented in Table 1.

**Table 1:** Biotechnological methods for the detection of antibiotic residues.

Type of Biosensor	Biorecognition Element	Transduction Mechanism	Key Advantages	Target Antibiotics	Scientific References
Enzyme-based	Enzymes (e.g., acetylcholinesterase)	Electrochemical (inhibition-based)	Fast, cost-effective	β-lactams, aminoglycosides	[28]
Immunosensor	Antibodies	Optical/electrochemical	High specificity, well-established	Tetracyclines, sulfonamides	[63]
Aptamer-based	DNA/RNA aptamers	Electrochemical or fluorescent	High affinity, reusability	Tetracyclines, chloramphenicol	[64]
Nanobiosensor	Antibodies or aptamers with nanoparticles	Enhanced electrochemical or optical signal	Increased sensitivity and stability	Various classes	[65]
Whole-cell sensor	Genetically modified bacteria	Luminescence or color change	Low cost, applicable in screening	General antibiotic activity	[66]

Despite their main advantages, biosensors still face challenges related to stability, reproducibility, and regulatory acceptance, which limit their widespread adoption in routine meat safety monitoring.

To further enhance biosensors’ performance, nanomaterials, such as nanoparticles and nanotubes, are being incorporated into biosensors to enhance their sensitivity, selectivity, and stability. These nanomaterials serve as catalytic agents, immobilization platforms, or optical and electroactive labels [28,64,65]. Among the various types, advanced electrochemical sensors stand out for their affordability, ease of use, and ability to detect multiple residues simultaneously. Their impedance-based measurements make them a practical choice for routine screening in meat production [66].

Future directions include the integration of biosensors with digital technologies, such as portable digital devices, wireless data transmission, and cloud-based analytics.

These advancements aim to facilitate real-time data collection, enhance traceability, and ensure more efficient monitoring of antibiotic residues throughout the food supply chain [61,67,68].

High-throughput screening is another powerful biotechnological tool that can be used to identify and evaluate potential solutions for antibiotic residue removal. This approach involves screening large libraries of enzymes, microorganisms, or other biological agents for their ability to degrade or bind to antibiotic molecules [58]. Compared to traditional culture-based methods, HTS significantly accelerates the discovery process by enabling parallel analysis of thousands of variants, reducing both time and labor.

A critical component of HTS’s success lies in the development of chromogenic substrates that are compatible with automated protocols. These substrates must support both qualitative and quantitative assessments, allowing researchers to attribute enzymatic activity to putative candidates and extract detailed information on enzyme properties [69]. When integrated into HTS workflows, such substrates enhance the precision and throughput of screening assays.

HTS methods have been effectively utilized to identify novel enzymes and microbial strains with improved degradation capabilities for specific antibiotics, including β-lactams, tetracyclines, and sulfonamides. These biological agents can be further optimized through protein engineering or adaptive evolution to function under industrial meat processing conditions. Moreover, HTS platforms are designed for scalability, making them suitable for industrial-scale applications including in-line processing systems and environmental remediation strategies, where large volumes of contaminated material must be treated efficiently [70,71,72].

Beyond its standalone capabilities, HTS can be synergistically integrated with biosensor technologies, which provide real-time monitoring of residue levels and enzymatic activity, enhancing both control and validation of remediation processes. Additionally, coupling HTS with genetic engineering approaches, such as directed evolution or CRISPR-based modifications, enables the refinement of microbial strains or enzymes for improved stability and performance under meat processing conditions [73,74].

Modern HTS platforms typically utilize microplate formats, automated liquid handling systems, and real-time optical or fluorescent readers to maximize accuracy and scalability. Despite these advantages, HTS methodologies also have their limitations. High setup costs, complexity of data interpretation, and the potential for false positives require rigorous assay optimization and validation protocols. Looking to the future, the integration of HTS with artificial intelligence and machine learning algorithms may further enhance its capabilities, allowing researchers to more efficiently identify and optimize enzymes or microbial strains suitable for the meat processing industry [75].

### 3.2. Biotechnological Methods for the Removal of Antibiotic Residues: Microbial, Enzymatic, and Engineered Strategies

Several biotechnological techniques are being developed and refined to remove antibiotic residues from meat and reduce antibiotic dependency. These include microbial degradation, enzymatic processes, bacteriophages, and genetically modified bacteria, as well as integrated treatment systems [76]. A summary of biotechnological methods for removing antibacterial residues and their mechanisms of action, applications, and advantages is shown in Table 2.

#### 3.2.1. Microbial Degradation

Microbial degradation is a promising approach for removing antibiotics from the enviroment. It involves the use of naturally occurring microorganisms, such as bacteria or fungi, which break down antibiotic molecules into less harmful substances [77,78]. This process often includes isolating and optimizing bacterial strains with specific enzyme systems capable of degrading a wide range of antibiotics [67]. These microorganisms can be applied in bioreactors or directly added to meat processing systems to reduce antibiotic residues at the source [79].

Environmental parameters such as temperature, pH, and nutrient availability have a significant impact on microbial metabolic rates and enzymatic activity. For instance, studies on tetracycline degradation have shown that optimizing these factors can greatly enhance degradation efficiency. In particular, iron-enhanced anaerobic digestion has demonstrated high removal rates of antibiotics such as sulfamethazine and sulfamethazole, with up to 86% degradation achieved for recalcitrant compounds like roxithromycin following the addition of zero-valent iron [80].

Biodegradation of antibiotics is usually a multistep process, starting with oxidation, reduction, or hydrolysis, followed by conjugation of these products with polar molecules such as sugars or amino acids, which improves solubility and facilitates further degradation [81]. Among the various environmental applications, microbial biodegradation plays a particularly important role in agriculture. For example, it is considered the primary mechanism for the elimination of antibiotics from swine wastewater, with reported removal rates exceeding 60% for veterinary antibiotics [82]. This highlights the key role of microbial communities in mitigating antibiotic pollution in agriculture. In addition, co-metabolism, where microorganisms degrade antibiotics in the presence of an additional growth substrate, can significantly increase efficiency, especially when low antibiotic concentrations are involved [82]. However, the success of these pathways is closely linked to environmental conditions. Parameters such as pH, temperature, and co-substrate availability directly determine the kinetics and completeness of the degradation reactions [83,84].

Anaerobic digestion has shown particular promise in the removal of pharmaceutical residues, with thermophilic conditions outperforming mesophilic and psychrophilic conditions, often doubling the degradation rate for several classes of antibiotics [85]. The efficacy of microbial degradation is also influenced by the composition of the microbial community, the physicochemical properties of the antibiotics involved, and operational parameters. These factors are critical for the success of advanced bioremediation strategies such as bio-attenuation, biostimulation, and bioaugmentation [86]. Compared to single-strain approaches, microbial consortia offer broader enzymatic diversity and greater resilience to environmental fluctuations, thereby improving degradation efficiency [86,87]. For instance, acclimated microbial sludge has been shown to enhance biodegradation within microbial fuel cells, accelerating co-substrate oxidation and the breakdown of compounds such as sulfamethoxazole [88]. Nevertheless, effective implementation requires precise control over system parameters such as inoculum type, pH, and temperature. Poorly optimized conditions can lead to incomplete degradation and the formation of toxic intermediates [86]. In certain contexts, anaerobic digestion has proven more effective than aerobic methods, particularly in treating dehydrated sludge [89]. However, extremely low initial concentrations of antibiotics can interfere with microbial activity, as seen with compounds like sulfamethoxazole, where minimal substrate availability limits degradation potential [90]. To address such limitations, advanced microbial electrochemical technologies, such as fluidized bed reactors, have emerged as effective tools for enhancing microbial degradation of persistent pollutants, even at trace concentrations [90]. One of the critical challenges in microbial degradation remains the complexity of antibiotic mixtures. Synergistic or antagonistic interactions between co-existing compounds can alter individual degradation pathways and affect overall removal efficiency [91].

Despite its potential, microbial degradation faces several limitations. The efficiency of degradation is highly dependent on environmental conditions such as pH, temperature, nutrient availability, and co-substrates. Extremely low concentrations of antibiotics can reduce microbial activity, limiting potential degradation. The complexity of antibiotic mixtures can lead to synergistic or antagonistic interactions, altering degradation pathways. Additionally, incomplete degradation may produce toxic intermediates, and poorly optimized systems can result in suboptimal removal. The deployment of microbial consortia or genetically modified strains also raises biosafety concerns, including horizontal gene transfer and unintended ecological impacts, which necessitate careful monitoring and risk assessment [74,79]. Finally, while microbial degradation offers significant potential, elevated concentrations of antibiotics can paradoxically inhibit bacterial activity, disrupt microbial processes, and potentially contribute to the development of antibiotic resistance [92]. Careful system design and ongoing monitoring are therefore essential to balance degradation efficiency with microbial ecosystem stability.

#### 3.2.2. Enzymatic Processes

Enzymatic degradation represents a highly specific and efficient strategy for neutralizing antibiotic residues. This approach involves the use of isolated enzymes that cleave specific chemical bonds within antibiotic molecules, thereby disrupting their structure and inactivating their biological function. One notable example is enzymes with antibacterial activity, enzybiotics, which offer promising alternatives to conventional antibiotics in the control of foodborne pathogens. These enzymes can be integrated into industrial processes for food safety and residue mitigation [93].

Enzymes used for degradation are typically derived from various sources, including bacteria, fungi, and plants. Through protein engineering, their stability and catalytic activity can be improved to withstand diverse environmental conditions, making them suitable for applications in food processing systems and wastewater treatment facilities [94,95]. For instance, hydrolase enzymes from activated sludge bacteria have shown the ability to degrade up to 70% of tetracycline, the most commonly used antibiotic in veterinary medicine [96]. The key advantages of enzymatic treatments lie in their high substrate specificity and mild operational conditions, which are particularly favorable for applications involving sensitive food matrices. Unlike broader biological systems, enzymes offer controlled reaction pathways that minimize the formation of undesirable byproducts.

Enzymatic degradation plays a crucial role in reducing the ecological impact of persistent antibiotic residues. These processes transform harmful compounds into less active or non-toxic derivatives, decreasing their potential to contribute to environmental antibiotic resistance. However, a critical consideration in enzymatic treatments is the fate of transformation products. Complete mineralization is not always achieved, and the resulting byproducts may still pose ecotoxicological risks. As such, comprehensive toxicological assessments are essential to evaluate the safety of enzymatic processes and ensure that the treatment does not inadvertently create new environmental hazards [97].

Despite their specificity and efficiency, enzymatic degradation strategies face several limitations. Enzyme stability under varying environmental conditions, incomplete mineralization of antibiotic residues, and the potential formation of toxic byproducts remain critical challenges. Moreover, large-scale application is often constrained by production costs and the need for precise control of operational parameters. Finally, as highlighted by Veiga-Crespo & Villa et al., the half-life, stability, and effectiveness of enzymes (enzybiotics) require further investigation before broad implementation in food processing or environmental applications [93].

#### 3.2.3. Genetically Modified Bacteria

Genetic engineering has significantly advanced microbial degradation strategies by enabling the insertion of genes encoding antibiotic-degrading enzymes into bacterial hosts. This genetic modification enhances both the efficiency and substrate range of microbial degradation, allowing for more effective breakdown of a variety of antibiotic compounds, including recalcitrant ones [80]. Such genetically engineered strains, often employed in mixed microbial consortia, demonstrate superior catabolic capabilities compared to single strains, benefiting from broader enzymatic diversity and increased resilience to environmental fluctuations [80,86,87].

Beyond whole-cell modifications, genetically engineered enzymes have also shown great promise. A notable example is LysK∆amidase, a modified phage lysin with potent antimicrobial activity against multidrug-resistant (MDR) bacteria such as MRSA. This enzyme not only disrupts resistant biofilms but also exhibits higher efficacy than some conventional antibiotics, positioning it as a promising tool in the fight against antibiotic-resistant pathogens [98].

Genetically modified bacteria can serve dual functions in bioremediation: they express antibiotic-degrading enzymes and can directly bind or remove antibiotic residues from environmental matrices. Such engineered strains have potential applications in meat processing and agricultural waste management, contributing to a reduction in antibiotic residues at their source [78]. Furthermore, the in situ production of antimicrobial peptides and endolysins by genetically modified organisms, either within animal feed or directly on food products, offers an innovative approach to enhance antibacterial effects while minimizing antibiotic use [99]. However, the deployment of genetically modified bacteria raises biosafety concerns, particularly regarding the horizontal gene transfer of engineered traits to indigenous microbial communities. This necessitates stringent ecological risk assessments and the development of robust containment strategies to mitigate unintended environmental impacts [100].

In addition to enzyme and strain engineering, CRISPR/Cas systems represent a cutting-edge genetic tool designed for targeted gene drives to reduce populations of antibiotic-resistant bacteria. By precisely editing resistance genes within microbial communities, this technology holds promise for directly addressing the root causes of antibiotic residue accumulation and resistance dissemination in food production systems [101,102]. The integration of such advanced biotechnologies into meat processing and environmental management frameworks offers a strategic avenue for safeguarding public health and combating the growing challenge of antimicrobial resistance [102,103].

Genetically modified bacteria enhance the efficiency and substrate range of microbial degradation, but their deployment faces several critical limitations. Biosafety concerns, particularly the potential for horizontal gene transfer of engineered traits to native microbial populations, remain a major challenge. Regulatory hurdles and public acceptance issues further complicate their implementation in food and agricultural systems. The survival, activity, and stability of engineered strains can be affected by environmental conditions, limiting their consistent efficacy. Additionally, the development and maintenance of these organisms are resource-intensive, and unintended ecological impacts on native microbial communities may arise. Addressing these challenges requires careful risk assessment, robust containment strategies, and ongoing monitoring to ensure safe and effective application [67].

#### 3.2.4. Phage Therapy and Biocontrol

Phage therapy represents a targeted and highly specific biological approach to combating bacterial infections and controlling antibiotic residues in food production systems. Bacteriophages, viruses that infect and lyse bacteria, offer a self-replicating antimicrobial strategy with a reduced likelihood of inducing resistance compared to traditional antibiotics [104,105,106,107]. Their application extends across various stages of the food production chain, effectively preventing pathogen colonization and biofilm formation, particularly on fresh produce and meat products [108,109,110].

Endolysins, hydrolytic enzymes derived from bacteriophages, are a key component of this strategy. These enzymes degrade the cell walls of Gram-positive bacteria rapidly and with high specificity, proving effective against antibiotic-resistant strains and biofilms [77,108,109,110]. Novel synthetic endolysins are being engineered to broaden their host range, including targeting Gram-negative bacteria, by modifying their structural domains to enhance bactericidal efficacy [111].

The commercial application of bacteriophages and their enzymes is growing in the field of food safety, supported by new regulatory frameworks designed to accommodate their biological complexity and the ability to monitor their efficacy [94,96]. In addition, the ability of phage therapy to reduce pathogen burden while sparing beneficial microbiota positions it as a promising alternative to broad-spectrum antibiotics in animal husbandry and food preservation [104,105,106,107,112].

Despite their promise, phage therapy and phage-derived biocontrol strategies face several limitations. The specificity of bacteriophages can limit their spectrum of action, requiring tailored cocktails for different bacterial strains. Environmental factors, including pH, temperature, and food matrix composition, can affect phage stability and efficacy. There is also a risk of bacterial resistance to phages over time, and regulatory approval processes for phage applications remain complex and region-specific. Moreover, large-scale production, formulation, and storage of phages and endolysins present practical challenges for consistent application in food systems [107,108].

#### 3.2.5. Phytoremediation

Phytoremediation represents a cost-effective and environmentally friendly strategy for removing antibiotic residues, particularly in agricultural and wastewater systems [113]. Certain plants and photosynthetic organisms, such as cyanobacteria, can degrade antibiotics while using light as an energy source and carbon dioxide as a carbon source, contributing simultaneously to carbon sequestration. This approach leverages the metabolic pathways of plants and microorganisms to transform or accumulate contaminants, making it a viable option for decontaminating meat production wastewater and other agricultural effluents. Microalgae further contribute through biodegradation, bioadsorption, and bioaccumulation, with intracellular metabolism and extracellular enzymatic activity driving effective removal of antibiotics such as florfenicol, reaching up to 86.67% removal efficiency in controlled systems [113,114].

Despite its environmental benefits, phytoremediation faces several limitations. The process is inherently slow, as it relies on plant growth and metabolic activity, which can take weeks to months to achieve significant reduction in antibiotic residues. Its effectiveness is highly dependent on environmental conditions such as light availability, temperature, nutrient levels, and water quality. Moreover, there is a risk that antibiotics or their metabolites may accumulate in plant tissues, potentially entering the food chain if plants are consumed or improperly disposed of. Seasonal variations and limited tolerance of plants to extreme conditions can further reduce the efficiency and consistency of this approach [113].

**Table 2:** Biotechnological methods for the removal of antibiotic residues.

Method	Mechanism of Action	Application	Key Advantages	Key Limitations and Challenges	References
Microbial degradation	Microorganisms enzymatically degrade antibiotics into less harmful compounds	Bioreactors, wastewater treatment, meat processing	Natural, scalable, eco-friendly	Sensitivity to environmental conditions; low substrate concentrations reduce efficiency; complex antibiotic mixtures alter degradation pathways; formation of toxic intermediates; biosafety concerns	[73,79]
Enzymatic degradation	Enzymes cleave chemical bonds in antibiotic molecules, rendering them inactive	In-line processing, enzyme additives	Specificity, rapid action, industrial scalability	Limited stability; incomplete degradation; high production cost	[67]
Genetically modified bacteria	Engineered strains produce degradation enzymes or bind antibiotics	Bioremediation, residue cleanup in processing facilities	Targeted action, customizable traits	Biosafety concerns; regulatory and public acceptance issues; environmental stability; complexity and cost	[93]
Bacteriophage therapy	Phages infect and lyse specific bacteria that carry antibiotic residues or resistance	Surface decontamination, animal gut microbiota control	Host specificity, minimal resistance development	Narrow host range/specificity; sensitivity to environmental conditions; risk of bacterial resistance development; regulatory and production challenges	[107,108]
Antimicrobial peptides	Peptides disrupt bacterial membranes or essential functions	Feed additives, infection control	Natural alternative to antibiotics	High production cost; susceptibility to proteolytic degradation; potential cytotoxicity at high doses	[59]
Phytoremediation	Plants absorb or transform antibiotic residues from the environment	Environmental cleanup near livestock areas	Low cost, sustainable	Slow remediation rates; strong environmental dependence; risk of antibiotic accumulation in plants	[113]
Biocatalysis	Microbial enzymes transform antibiotic residues into non-toxic products	Combined with other strategies	Eco-friendly, highly adaptable	Limited substrate range; sensitivity to environmental conditions; potential formation of toxic byproducts	[115]
Integrated AOP + bioremediation	AOPs break down complex molecules; microbes further degrade intermediates	Sequential wastewater or surface treatment	Synergistic, more complete degradation	High operational complexity and cost; potential interactions reducing efficiency; variability in performance due to environmental and feedstock differences; challenges in industrial-scale implementation	[115]

#### 3.2.6. Biocatalysis

Biocatalysis utilizes enzymes or whole-cell biocatalysts to degrade antibiotic residues efficiently, often under mild operational conditions. This approach can be integrated into wastewater treatment or environmental remediation workflows and benefits from the specificity and catalytic activity of enzymes derived from bacteria, fungi, or metagenomic sources [115]. Biocatalytic systems can target a wide range of antibiotics and are increasingly investigated for scalable, eco-friendly solutions in food production and agricultural waste management.

Biocatalytic approaches, while highly specific and efficient under controlled conditions, also face challenges. The substrate range of enzymes can be limited, meaning that not all antibiotics are effectively degraded. Enzyme stability and catalytic activity are sensitive to environmental parameters such as pH, temperature, and the presence of inhibitors or contaminants in wastewater. Additionally, incomplete degradation may result in the formation of transformation products that could still possess biological activity or ecotoxicity. Scaling up biocatalytic processes from the laboratory to industrial applications requires careful optimization and monitoring to maintain consistent performance [115].

#### 3.2.7. Integrated Approaches

Integrated approaches use a synergistic combination of multiple remediation strategies to improve antibiotic removal efficiency beyond that possible with any single method. For example, coupling advanced oxidation processes with bioremediation techniques allows the breakdown of complex antibiotic molecules into more bioavailable intermediates, which can then be efficiently degraded by microorganisms [116]. By applying such methods, either sequentially or in parallel, within food processing workflows, these integrated systems offer promising results in reducing antibiotic residues while maintaining operational feasibility. Furthermore, the integration of antimicrobial peptides and endolysins—whether natural or synthetically enhanced—into livestock management or food processing frameworks provides a powerful complementary strategy. These biomolecules function with high specificity and without reliance on traditional antibiotics, thereby helping to suppress the development of resistance [103]. When implemented together, these strategies contribute to meeting the growing global demand for animal protein while simultaneously addressing the spread of antibiotic resistance in the food supply chain [117,118].

Integrated approaches offer synergistic benefits, but they also present several limitations. The complexity of combining multiple remediation strategies can increase operational costs and require advanced technical expertise to optimize process parameters. Interactions between different components, such as enzymes, microorganisms, or chemical oxidants, may lead to reduced efficiency or formation of unintended byproducts. Additionally, variability in wastewater composition, antibiotic concentrations, and environmental conditions can affect the reproducibility and overall performance of these integrated systems. Finally, scaling up from laboratory or pilot setups to industrial-scale implementation remains challenging, demanding careful monitoring and control to ensure consistent antibiotic removal [116].

### 3.3. Enabling Technologies for Antibiotic Residue Mitigation: Genomic, Computational, and Monitoring Tools

Available new technologies form the backbone of modern biotechnological strategies aimed at mitigating antibiotic residues in livestock and food systems. Genomic, computational, and monitoring tools not only allow precise detection of residues and resistance genes, but also support the design and control of advanced bioprocesses. For instance, enzyme immobilization and reactor design strategies improve the stability and reusability of biocatalysts, thereby enhancing the economic feasibility of enzymatic degradation at industrial scales [119]. Complementing these approaches, microbial electrochemical systems—such as fluidized bed reactors—stimulate microbial metabolic pathways, enabling effective removal even of trace-level pollutants [90].

Metagenomics-based surveys of food-system microbial communities facilitate the tracking of antibiotic resistance gene dissemination from farm environments, offering a powerful tool for surveillance and risk mitigation [120]. Combined with computational analyses, these molecular techniques enable real-time monitoring of microbial adaptation under selective pressures. They also assist in uncovering novel gene-editing targets for refined interventions in antimicrobial resistance management [121,122].

#### 3.3.1. Metagenomics and Bioinformatics

Metagenomics, in combination with advanced bioinformatic tools, provides a comprehensive framework for analyzing microbial communities in animal production environments. These approaches are instrumental in identifying the sources and mechanisms of antibiotic persistence, as well as tracking AMR. Through high-throughput sequencing, metagenomic methods enable the detection of antibiotic-resistant genes and allow profiling of microbial taxa associated with antibiotic degradation and survival [123]. Bioinformatics plays a crucial role in managing and interpreting complex genomic datasets, facilitating the tracking of resistance gene evolution, and designing targeted microbial or enzymatic interventions. These insights support the development of precision strategies to reduce antibiotic contamination, contributing to sustainable livestock management and enhanced food safety. Moreover, the integration of metagenomics enables the selection of optimal microbial consortia and environmental conditions for interventions, while preserving the quality of food products [111]. This is particularly relevant when selecting strains for biodegradation or biocontrol that will not adversely affect sensory or nutritional properties.

Advances in metagenomics have led to the discovery of novel enzymatic pathways from complex microbial communities. These pathways, often previously unknown, contribute to the degradation or transformation of persistent antibiotic residues into less toxic or inactive byproducts, reducing their ecological footprint [82,121]. Thus, metagenomic analysis not only aids in surveillance but also expands the enzymatic toolbox for antibiotic detoxification.

#### 3.3.2. Detection of Antibiotic Resistance Genes

Accurate detection of antibiotic resistance genes is essential for monitoring and controlling antibiotic residues. Techniques like PCR and next-generation sequencing are widely used for rapid identification of resistance genes in bacterial isolates from food and livestock. Advances in microfluidic devices further enhance detection sensitivity and speed, making them suitable for on-site applications. Additionally, metagenomic analysis of environmental samples provides a comprehensive overview of the diversity, distribution, and expression of resistance genes, offering valuable insights to inform effective mitigation strategies. Together, these genomic and molecular tools form the foundation for understanding and managing the spread of antibiotic resistance in agricultural and food production systems [124].

#### 3.3.3. Rational Design and Engineering

Rational design and protein engineering are key approaches which can improve enzymes and microbial systems for more efficient antibiotic degradation. Utilizing advanced biotechnological tools such as site-directed mutagenesis, fusion protein construction, and surface display systems enables the precise modification of enzyme structures to meet specific industrial requirements [125]. Additionally, genome-editing tools like CRISPR/Cas9 and synthetic biology platforms offer fine-tuned control over microbial strains, facilitating the development of biocatalysts with improved stability and activity under processing conditions [126]. Enzymes derived from extremophiles, microorganisms thriving in extreme environments, present a vast array of industrial applications due to their remarkable tolerance to harsh conditions [127]. Moreover, a comprehensive understanding of microbial metabolism supports the conversion of agricultural byproducts and food wastes into nutritionally enhanced products, contributing to both environmental sustainability and food-system innovation [128].

#### 3.3.4. Computational Tools and Modeling

Computational technologies play an increasingly vital role in the development and optimization of biotechnological strategies for antibiotic residue mitigation. Metabolic pathway modeling and enzyme activity simulations are essential for designing synthetic microbial consortia and guiding metabolic engineering interventions. These models facilitate a deeper understanding of microbial interactions and support the rational selection of strains and enzymes with enhanced bioremediation potential. Moreover, machine learning (ML) and artificial intelligence (AI) algorithms are being integrated into protein engineering workflows to predict enzyme–substrate interactions, guide mutagenesis strategies, and streamline variant selection processes. Automation tools further contribute by enabling high-throughput optimization of biocatalysts and synthetic metabolic pathways, thereby reducing the experimental burden and accelerating the development of effective solutions [115,129].

### 3.4. Biotechnological Applications and Case Studies

The practical application of biotechnological innovations has demonstrated significant promise in reducing antibiotic residues within livestock production systems. For example, the use of direct-fed microbials, such as probiotics and prebiotics, has been shown to enhance gut health and immune response in animals, thereby decreasing the need for antibiotic interventions [59]. In addition, bacteriophage therapy has proven effective in lowering bacterial contamination, including successful reduction in *Salmonella* levels in poultry products. The study by Atterbury et al. investigated whether bacteriophages can reduce Salmonella contamination on chicken skin. After experimentally infecting broiler chickens, skin sections were treated with specific Salmonella phages. Phage treatment significantly reduced Salmonella counts on chicken skin by 1.38 log_10_ MPN for S. Enteritidis and 1.83 log_10_ MPN for S. Typhimurium compared to controls [130]. Another promising approach involves the deployment of microbial consortia capable of degrading antibiotic compounds like tetracycline in animal waste, which not only mitigates environmental contamination but also supports circular and sustainable agricultural practices. These applications collectively illustrate the broad utility of biotechnology in reducing antibiotic reliance and minimizing its ecological and public health impacts.

### 3.5. Monitoring and Detection Technologies

The successful implementation of biotechnological interventions necessitates reliable and sensitive monitoring systems to ensure both efficacy and safety. Advanced biosensors, along with analytical methods such as liquid chromatography–mass spectrometry (LC-MS), provide accurate and rapid detection of antibiotic residues in meat and environmental samples [131,132,133]. Real-time monitoring capabilities allow for immediate intervention during processing, reducing the risk of contaminated products entering the food chain [134,135,136]. Furthermore, comprehensive data analysis plays a critical role in assessing the effectiveness of applied biotechnological solutions, offering valuable insights for continuous process improvement and regulatory compliance [132,133,137].

## 4. Risk Assessment and Public Acceptance of Biotechnology in Meat Production

The application of biotechnological interventions in meat production brings forth important safety, ethical, and societal considerations. This includes potential risks to animal health, environmental impacts, and the unintended consequences of genetic modifications or microbial interventions. A comprehensive risk assessment is essential for to evaluate the implementation of biotechnological solutions into the meat industry. Transparent communication strategies and education are needed to build trust and address societal concerns, especially in light of historical apprehensions surrounding genetically modified organisms [138].

Addressing AMR is central to the justification of biotechnology use in livestock. The growing threat of AMR demands alternatives to antibiotic use as growth promoters. Approaches such as phage therapy, microbial degradation, enzymatic breakdown, and CRISPR/Cas9 applications offer innovative avenues for antibiotic residue mitigation. However, each of these presents its own challenges. For instance, while phages are promising due to their specificity and minimal disruption to the microbiome, they may contribute to bacterial resistance evolution and immune responses in the host [139,140]. Likewise, CRISPR-based antimicrobials require further refinement for effective and broad application [141]. In addition, biostimulation and bioaugmentation strategies, employing indigenous or introduced microorganisms to degrade antibiotics, hold great promise. However, these require optimization and validation for practical use in food systems. Enzymatic treatments, often used in tandem with microbial processes, also face hurdles related to stability, substrate specificity, and scalability [142]. Ultimately, all technological advancements must align with ethical principles, ensure equitable access to safe food, and support environmental sustainability.

Regulatory complexity remains a major barrier to implementation, as novel biotechnological products must pass rigorous safety assessments before approval for use in food production [143]. This process, while essential, can delay the adoption of promising interventions. Cost-effectiveness is also a challenge, especially for low- and middle-income countries where advanced technologies may not be readily available. To ensure equitable implementation, public policies need to promote accessibility, while continued scientific validation must address concerns about environmental impacts, unintended gene transfer, and long-term safety. A balanced approach that integrates innovation, regulation, and public trust is essential for the successful application of biotechnology in meat production.

## 5. Challenges and Future Directions for Biotechnological Applications in Antibiotic Residue Reduction

Future work should prioritize international regulatory harmonization, particularly for genetically modified microorganisms, nano-enabled detection systems, and RNA-based interventions used in food processing, as inconsistent approval frameworks remain a major barrier to commercialization [12,143]. Establishing unified global frameworks would streamline approval pathways, reduce duplication of safety assessments, and accelerate the deployment of biotechnology across different markets [144]. To improve scalability, research should also focus on cost-reduction strategies, such as enzyme immobilization to enable repeated use, development of low-cost bioreactors for microbial biosensors, and optimization of fermentation-based production to decrease manufacturing expenses [143]. In parallel, risk-assessment models tailored to biotech interventions in meat processing are needed to evaluate environmental dispersion, horizontal gene transfer, and long-term ecological impact with higher precision than existing tools [12,145].

Advancing these solutions will also require large-scale validation studies directly within slaughterhouses and meat processing facilities to assess real-world performance, robustness, and integration with existing quality-control systems [144]. Future directions should further include the creation of interoperable digital platforms that link biosensor outputs, microbial surveillance data, and AMR profiles across supply chains, enabling predictive monitoring and rapid response [2,145]. Finally, strengthening One Health-based global surveillance will be essential to quantify the contribution of foodborne pathways to AMR transmission and to guide targeted interventions where they are most needed [2,12].

## 6. Conclusions

Biotechnology offers a promising solution to improve meat safety by effectively removing antibiotic residues and reducing the risk of antibiotic resistance transmission. The combined use of genomic, enzymatic, microbial, and computational technologies enables targeted, efficient, and potentially sustainable solutions to this global challenge. Equally important is the role of computational modeling and real-time monitoring technologies, which are the basis of modern strategies for mitigating antibiotic residues. These approaches not only improve detection, characterization, and degradation processes, but also facilitate the development of more effective and scalable interventions. By bridging analytical precision with biotechnological innovation, these approaches significantly contribute to safer food production systems and broader efforts to combat antimicrobial resistance. However, these strategies must be developed and implemented within a framework that ensures public trust, ethical accountability, and regulatory compliance.

As traditional antibiotics lose their effectiveness, biotechnological innovations offer a critical alternative path and a leap forward. However, the ease of gene transfer among bacteria, including foodborne pathogens, requires careful monitoring and strategic planning. A multi-pronged approach that integrates responsible antibiotic use, cutting-edge biotechnology, and strong public health policies will be necessary in the future. Only through such coordinated efforts can we ensure the continued safety of food products, protect global health, and mitigate the growing threat of antimicrobial resistance.

## Data Availability

The original contributions presented in the study are included in the article. Further inquiries can be directed to the corresponding author.
